# Ultrasound examination supporting CT or MRI in the evaluation of cervical lymphadenopathy in patients with irradiation-treated head and neck cancer

**DOI:** 10.1515/med-2023-0682

**Published:** 2023-04-12

**Authors:** Ping-Chia Cheng, Chih-Ming Chang, Li-Jen Liao, Chen-Hsi Hsieh, Pei-Wei Shueng, Po-Wen Cheng, Wu-Chia Lo

**Affiliations:** Department of Otolaryngology Head and Neck Surgery, Far Eastern Memorial Hospital, New Taipei City 220, Taiwan (R.O.C.); Head and Neck Cancer Surveillance and Research Study Group, Far Eastern Memorial Hospital, New Taipei City, Taiwan (R.O.C.); Graduate Institute of Medicine, Yuan Ze University, Taoyuan, Taiwan (R.O.C.); Department of Communication Engineering, Asia Eastern University of Science and Technology, New Taipei City, Taiwan (R.O.C.); Department of Biomedical Engineering, National Yang-Ming University, Taipei, Taiwan (R.O.C.); Department of Electrical Engineering, Yuan Ze University, Taoyuan, Taiwan (R.O.C.); Medical Engineering Office, Far Eastern Memorial Hospital, New Taipei City, Taiwan (R.O.C.); Division of Radiation Oncology, Department of Radiology, Far Eastern Memorial Hospital, Taipei, Taiwan; Department of Medicine, School of Medicine, National Yang-Ming University, Taipei, Taiwan (R.O.C.); Department of Otolaryngology Head and Neck Surgery, Far Eastern Memorial Hospital, No. 21, Sec. 2, Nanya S. Rd., Banqiao Dist., New Taipei City 220, Taiwan (R.O.C.)

**Keywords:** lymphadenopathy, ultrasound, fine‐needle aspiration cytology, computed tomography, magnetic resonance imaging

## Abstract

In this study, we determined the diagnostic performance of adding ultrasound (US) with/without fine‐needle aspiration cytology (FNAC) to computed tomography (CT)/magnetic resonance imaging (MRI) in evaluating neck lymphadenopathy (LAP) in patients with head and neck cancer treated with irradiation. We included 269 patients who had neck LAP after radiotherapy (RT) or concurrent chemoradiotherapy (CCRT) resulting from cancers of the head and neck region between October 2008 and September 2018. The diagnostic methods consisted of the following: 1) CT/MRI alone, 2) CT/MRI combined with a post-RT US predictive model, and 3) CT/MRI combined with US + FNAC. We compared their diagnostic performance using receiver operating characteristic (ROC) curves. In total, 141 (52%) malignant and 128 (48%) benign LAPs were observed. Regarding the diagnostic accuracy, the area under the ROC curves was highest for the combined CT/MRI and US + FNAC (0.965), followed by the combined CT/MRI and post-RT US predictive model (0.906) and CT/MRI alone (0.836). Our data suggest that the addition of a US examination to CT/MRI resulted in higher diagnostic performance than CT/MRI alone in terms of diagnosing recurrent or persistent nodal disease during the evaluation of LAP in patients with irradiation-treated head and neck cancer.

## 1 Introduction

Radiotherapy (RT) is the standard treatment for advanced stage head and neck cancer. However, following neck irradiation, the surveillance of nodal malignancy is challenging. RT may result in tissue fibrosis, increasing the difficulty of palpating an enlarged node [1,2]. The lymph node (LN) recurrence rate of head and neck cancer after RT ranges from <10 to 29% [2,3]. Magnetic resonance imaging (MRI) or computed tomography (CT) is typically applied to detect such recurrence [4], although ultrasound (US) has been gradually introduced for more precise detection [5,6]. The 2020 National Comprehensive Cancer Network (NCCN) guidelines report that US, CT, MRI, and positron emission tomography (PET)/CT have distinct advantages in the posttreatment follow-up of patients with locoregionally advanced head and neck cancer [7]. The follow-up intervals for CT, MRI, and PET/CT are clearly defined in the 2020 NCCN guidelines. However, US is typically used as an adjuvant imaging tool, and its role is less emphasized. In this study, we explored whether adding US with or without fine‐needle aspiration cytology (FNAC) to CT/MRI improves diagnostic accuracy. Studies comparing the diagnostic rate of US and CT/MRI during the posttreatment evaluation of patients with head and neck cancer remain limited. Investigating patients with nasopharyngeal carcinoma (NPC), Toh et al. reported that the positive predictive value (PPV) of recurrent nodal metastasis was 93.8% for FNAC and 78.6% for CT during posttreatment follow-up [8]. In patients with head and neck cancer who had completed concurrent chemoradiotherapy (CCRT), Nishimura et al. observed that the sensitivity and specificity of diagnosing malignant LN were 52.9 and 74.2% for CT/MRI, 88.2 and 66.1% for US, and 71.4 and 95.6% for FNAC, respectively [9]. In our previous study, we developed a post-RT US predictive model for the prediction of recurrent or persistent nodal disease in irradiation-treated patients [10]. The model was 1.35 × (long axis) + 2.03 × (short axis) + 2.27 × (margin) + 1.48 × (echogenic hilum) + 3.7. If the score was equal to or greater than 7, an LN was regarded as malignant. This predictive model exhibited favorable sensitivity, PPV, negative predictive value (NPV), and accuracy (85, 82, 83, and 83%, respectively). In this study, we explored the effect of adding US with or without FNAC to CT/MRI in the assessment of recurrent or persistent lymphadenopathy (LAP) in patients with irradiation-treated head and neck cancer under a retrospective setting. Furthermore, we compared the diagnostic performance of CT/MRI alone, CT/MRI in combination with the post-RT US predictive model, and CT/MRI in combination with US + FNAC.

## 2 Materials and methods

### 2.1 Inclusion and exclusion criteria

**Figure 1:**
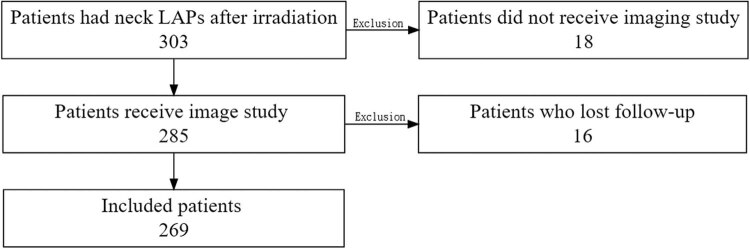
Flow chart of study inclusion and exclusion criteria.

This retrospective study was performed at a tertiary medical center. The reporting of the study followed the Standards for Reporting Diagnostic accuracy studies (STARD) statement. Data from patients who received RT or CCRT for the treatment of cancers in the head and neck region between October 2008 and September 2018 were reviewed. Patients who had LAP, which was defined as the presence of one or many LN(s) detected through a palpation or imaging study, after neck irradiation were included in this study. We included both patients who had or did not have previous neck dissection. All patients received either CT or MRI together with US with or without FNAC approximately 2–3 months after RT/CCRT, followed by every 6 months or under suspicion of recurrence. The CT or MRI was performed with contrast under a 3 mm or 5 mm slice, respectively. Usually, we arranged MRI for the surveillance. If patients were intolerant to MRI examination due to claustrophobia, dyspnea, or not suitable for the prolonged supine position, we arranged CT for the evaluation. US was performed using the brightness and Doppler mode without contrast. Ultrasound-guided fine-needle aspiration (USgFNA) was performed in patients exhibiting suspicious US features during examination [11]. If a patient had one or multiple LNs with suspicion of malignancy, we chose the largest LN for USgFNA. Patients who were lost to follow-up after neck irradiation or did not undergo an imaging study were excluded (Figure 1).

The final diagnoses were obtained according to either clinical diagnoses after multidisciplinary discussions or pathological diagnoses through means of core needle biopsy, excisional biopsy, or neck dissection. In clinical diagnosis, an LAP was regarded as benign when no size change was observed during the course of 12-month follow-up; an LAP was considered malignant when the disease was clearly observed during the imaging study, which was verified with an abnormal cytological report (malignancy, suspicion of malignancy, or atypical cells), or when an LAP was observed to be obviously enlarged during further image study.

### 2.2 Clinical characteristics and outcome assessment

We recorded information on the age, gender, duration between RT/CCRT and the imaging study, primary malignancy, CT or MRI imaging, US examination, and FNAC report from the medical records. The short axis, long axis, and short-to-long axis (S/L) ratio of neck LN were documented from the US images. Nishimura et al. evaluated the ability of CT/MRI, US, or FNAC to diagnose malignant LNs [9]. However, in clinical practice, FNAC is seldom performed without US. Thus, in this study, the single diagnostic examinations analyzed were CT/MRI, the post-RT US predictive model, and US + FNAC. The diagnosis of CT/MRI was determined by an experienced radiologist based on the patient’s medical history. Following neck irradiation, an enhancement and expansion feature, such as an irregular margin, on an LN was regarded as a malignancy [12]. The post-RT US predictive model was that used in our earlier study [10]. If its score was ≥7, a node was considered to be malignant. The diagnosis of US + FNAC was mainly based on the cytological report, which was supported using the post-RT US predictive model. If the cytological report indicated the presence of malignancy, suspicion of malignancy, or atypical cells, this LN was regarded as malignant. If no cytological report was available, we used the post-RT US predictive model to determine LN malignancy or benignity.

### 2.3 Statistical analysis

A two-sample *t*-test was used for continuous variables, and the chi-squared or Fisher exact test was used for categorical variables. The odds ratio (OR) with a 95% confidence interval was reported. Other studies have only focused on the accuracy of single diagnostic examinations rather than that of combined methods. In our study, we evaluated the effect of adding a US examination to CT/MRI by assessing the following diagnostic methods: 1) CT/MRI alone, 2) CT/MRI combined with the post-RT US predictive model, and 3) CT/MRI combined with US + FNAC. According to the final diagnoses, we calculated the predicted probability for nodal malignancy using logistic regression. The diagnostic performance was also compared using receiver operating characteristic (ROC) curves and the area under the receiver operating characteristic curves (AUC). AUC differences by using paired-sample area differences under the ROC curves were also executed. Statistical significance was indicated if *p* < 0.05. Statistical analysis was conducted using SPSS software version 28 (IBM, Armonk, NY, USA).


**Ethical considerations:** This study was approved by the institutional ethical review board of Far Eastern Memorial Hospital (No. 109140-E). The study did not influence the patients’ treatment or outcome. All data were analyzed using a deidentified form; the data set is presented in the supplementary material (Table S1).

## 3 Results

A total of 269 patients who exhibited LAP following neck RT were included in our study (Table 1); these patients were predominantly men (85% [228 of 269]). The mean (standard deviation [SD]) age was 53 (10) years, and the mean (SD) duration between RT/CCRT and imaging study was 583 (763) days. According to the final diagnosis, the malignancy rate was 52%. We recorded 141 malignant LNs and 128 benign LNs. The most common primary sites were oral cancer, NPC, and hypopharyngeal cancer. Among these three primary malignancies, oral cancer (66% [61 of 93]) and hypopharyngeal cancer (62% [23 of 37]) had higher nodal malignancy rates and NPC (35% [30 of 85]) had lower malignancy rate. The clinical characteristics were compared according to the final diagnoses. We observed significant differences in age (*p* < 0.001), duration between RT/CCRT and imaging study (*p* = 0.04), short axis (*p* < 0.001), long axis (*p* < 0.001), and the S/L ratio (*p* < 0.001) but not gender (Table 1).

**Table 1:** Comparison of the clinical characteristics between the malignant and benign nodal diseases

Variables	ALL	Malignancy	Benignity		
No. (%) or mean (SD)	(*N* = 269)	(*N* = 141)	(*N* = 128)	Difference (95% CI)	*p*-value
Age, years	53 (10)	56 (10)	50 (11)	5.54 (3.11–7.96)	<0.001*
Gender				−7% (−24 to 9%)	0.398
Female	41 (15%)	19 (13%)	22 (17%)		
Male	228 (85%)	122 (87%)	106 (83%)		
Duration between RT/CCRT and imaging study, days	583 (763)	491 (676)	683 (840)	−192 (−374 to −9.88)	0.039*
Exams that patients received					
MRI	259 (96%)	133 (94%)	126 (98%)		
CT	10 (4%)	8 (6%)	2 (2%)		
US	269 (100%)	141 (100%)	128 (100%)		
FNAC	251 (93%)	140 (99%)	111 (87%)		
US image					
Short axis, cm	0.92 (0.54)	1.17 (0.57)	0.64 (0.33)	0.53 (0.42–0.64)	<0.001*
Long axis, cm	1.48 (0.82)	1.80 (0.93)	1.12 (0.48)	0.67 (0.49–0.85)	<0.001*
S/L ratio	0.64 (0.17)	0.68 (0.16)	0.59 (0.18)	0.09 (0.05–0.13)	<0.001*
Primary malignancy					
Oral cancer	93 (34%)	61 (43%)	32 (25%)		
NPC	85 (32%)	30 (21%)	55 (43%)		
Hypopharyngeal cancer	37 (14%)	23 (16%)	14 (11%)		
Oropharyngeal cancer	24 (9%)	8 (6%)	16 (12%)		
Laryngeal cancer	17 (6%)	12 (9%)	5 (4%)		
Parotid cancer	7 (3%)	2 (1%)	5 (4%)		
Unknown primary tumors of head and neck	3 (1%)	2 (1%)	1 (1%)		
Cervical esophageal cancer	1 (0.3%)	1 (1%)	0 (0%)		
Nasal malignant melanoma	1 (0.3%)	1 (1%)	0 (0%)		
Conjunctival cancer	1 (0.3%)	1 (1%)	0 (0%)		

Abbreviations: CT, computed tomography; FNAC, fine-needle aspiration cytology; LAP, lymphadenopathy; MRI, magnetic resonance imaging; RT/CCRT, radiotherapy/concurrent chemoradiotherapy; S/L, short-to-long axis; US, ultrasound.

*Statistical significance, *p* < 0.05.

The diagnostic examinations for assessing neck LAP were compared according to the final diagnoses (Table 2). We noted significant differences in distinguishing benign from malignant nodal disease in all three diagnostic exams (*p* < 0.001). The OR was highest in US + FNAC, followed by the CT/MRI report, and then the post-RT US predictive model (103.9, 30.1, and 28.4, respectively).

**Table 2:** Comparison of the diagnostic exams in assessing neck LAP in post-irradiation head and neck cancer patients

Diagnostic exam	Final diagnosis		OR (95% CI)	Difference (95% CI)	*p*-value
	Malignancy	Benignity			
CT/MRI			30.1 (15.0–60.4)	68% (56–80%)	<0.001*
As malignancy	109 (89%)	13 (11%)			
As benignity	32 (22%)	115 (78%)			
Post-RT US predictive model^†^			28.4 (14.3–56.3)	68% (56–80%)	<0.001*
As malignancy (score ≥7)	127 (80%)	31 (20%)			
As benignity (score <7)	14 (13%)	97 (87%)			
US + FNAC			103.9 (44.9–240.4)	82% (70–94%)	<0.001*
As malignancy	129 (91%)	12 (9%)			
As benignity	12 (9%)	116 (91%)			

Abbreviations: CT/MRI, computed tomography/magnetic resonance imaging; FNAC, fine-needle aspiration cytology; LAP, lymphadenopathy; RT, radiotherapy; US, ultrasound. ^†^It was composed of 1.35 × (long axis) + 2.03 × (short axis) + 2.27 × (margin) + 1.48 × (echogenic hilum) + 3.7. *Statistical significance, *p* < 0.05.

The predicted probability for malignancy for CT/MRI alone, the combined CT/MRI and post-RT US predictive model, and the combined CT/MRI and US + FNAC is presented in Table 3. For cases where the diagnostic exams revealed malignancy, the predicted probability for malignancy was highest in the CT/MRI and US + FNAC combination (0.988), followed by the CT/MRI and post-RT US predictive model combination (0.933), and CT/MRI alone (0.893).

**Table 3:** Predicted probability for malignancy among CT/MRI alone, the combined CT/MRI and post-RT US predictive model, and the combined CT/MRI and US + FNAC in the evaluation of neck LAP in post-irradiation head and neck cancer patients

CT/MRI result	Post-RT US predictive model result	US + FNAC result	Predicted probability for malignancy
**CT/MRI alone**			
Malignancy			0.893
Benignity			0.107
**Combined CT/MRI and post-RT US predictive model**			
Malignancy	Malignancy		0.933
Malignancy	Benignity		0.530
Benignity	Malignancy		0.509
Benignity	Benignity		0.077
**Combined CT/MRI and US + FNAC**			
Malignancy		Malignancy	0.988
Benignity		Malignancy	0.731
Malignancy		Benignity	0.440
Benignity		Benignity	0.026

Abbreviations: CT/MRI, computed tomography/magnetic resonance imaging; FNAC, fine-needle aspiration; RT, radiotherapy; US, ultrasound.

**Figure 2:**
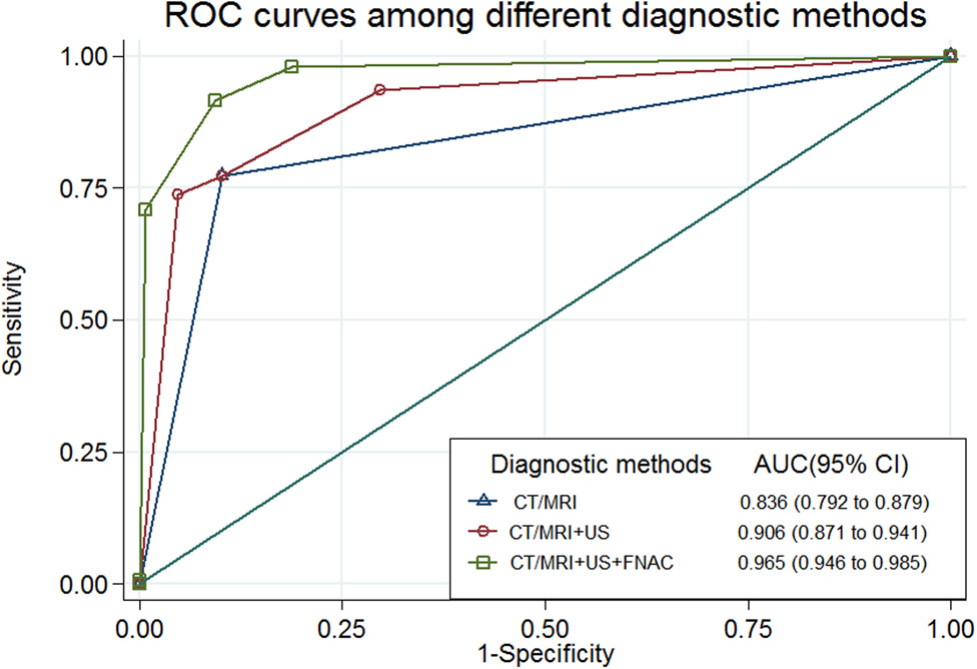
ROC curves for diagnosing nodal malignancy among CT/MRI alone, the combined CT/MRI and post-RT US predictive model, and the combined CT/MRI and US + FNAC. The AUC differences (95% CI) were −0.07 (−0.10 to −0.05) for CT/MRI vs CT/MRI + US, −0.13 (−0.17 to −0.09) for CT/MRI vs CT/MRI + US + FNAC, and −0.06 (−0.09 to −0.03) for CT/MRI + US vs CT/MRI + US + FNAC; all *p*-values < 0.001.

The ROC curves of CT/MRI alone, the combination of CT/MRI with the post-RT US predictive model, and the combination of CT/MRI with US + FNAC in the evaluation of nodal recurrence or persistence are illustrated in Figure 2. The AUC was highest in the combination of CT/MRI with US + FNAC (0.965), followed by the combination of CT/MRI with post-RT US (0.906), and then CT/MRI alone (0.836). We further compared the performance of three diagnostic methods by using paired-sample area difference under the ROC curves, and all AUC differences showed significant differences (AUC differences [95% CI], −0.07 [−0.10 to −0.05] for CT/MRI vs CT/MRI + US, −0.13 [−0.17 to −0.09] for CT/MRI vs CT/MRI + US + FNAC, and −0.06 [−0.09 to −0.03] for CT/MRI + US vs CT/MRI + US + FNAC, all *p*-values < 0.001). Table 4 summarizes the false-negative results if the LAP diagnosis was based on CT/MRI alone and on CT/MRI combined with US + FNAC during the follow-up period after completion of RT/CCRT. The false-negative rate was significantly higher in patients who were diagnosed using CT/MRI alone than that of patients diagnosed using combined CT/MRI and US + FNAC (11% [29/269] vs 1% [3/269], *p* < 0.001).

**Table 4:** False-negative results if the LAP diagnosis was based on CT/MRI alone and on the combined CT/MRI and US + FNAC

	False-negative cases			
Variables, No. (%) or mean (SD)	CT/MRI alone (*N* = 29)	Combined CT/MRI and US + FNAC (*N* = 3)	Difference (95% CI)	*p*-value
Incidence rate	29/269 (11%)	3/269 (1%)	10% (6–14%)	<0.001*
Age, years	57 (10)	56 (7)	1.20 (−10.36 to 12.75)	0.834
Gender			0.1 (0.0–8.39)^†^	0.181^†^
Female	1 (3%)	1 (33%)		
Male	28 (97%)	2 (67%)		
Duration between RT/CCRT and imaging study, days	423 (433)	498 (398)	−75 (−609 to 458)	0.775
US image				
Short axis, cm	1.08 (0.53)	0.46 (0.21)	0.62 (−0.01 to 1.25)	0.055
Long axis, cm	1.64 (0.76)	0.98 (0.36)	0.66 (−0.26 to 1.58)	0.154
S/L ratio	0.67 (0.13)	0.46 (0.04)	0.20 (0.05–0.36)	0.013*
Prior malignancy				
Oral cancer	13 (45%)	0 (0%)		
NPC	5 (17%)	3 (100%)		
Hypopharyngeal cancer	6 (21%)	0 (0%)		
Laryngeal cancer	2 (7%)	0 (0%)		
Unknown primary tumors of head and neck	2 (7%)	0 (0%)		
Nasal malignant melanoma	1 (3%)	0 (0%)		

Abbreviations: CT/MRI, computed tomography/magnetic resonance imaging; FNAC, fine needle aspiration cytology; RT/CCRT, radiotherapy/concurrent chemoradiotherapy; S/L, short-to-long axis; US, ultrasound. ^†^Fisher exact test with corresponding OR and 95 CI. *Statistical significance, *p* < 0.05.

## 4 Discussion

Although the improved survival rate for the early detection of malignancy may result from lead time bias, regular surveillance for nodal recurrence or persistence is still crucial during the follow-up period after primary treatment for head and neck cancer. Several studies have demonstrated that early detection of malignancy benefits the survival rate [5,13,14]. However, for patients with head and neck cancer who have undergone neck irradiation, clinicians may face difficulty in the evaluation of nodal disease because of tissue fibrosis [1,2]. In this study, we determined that for detecting nodal recurrence or persistence, the combined CT/MRI and post-RT US predictive model had a higher AUC than that of CT/MRI alone (0.906 vs 0.836; Figure 2). Moreover, we observed that the combined CT/MRI and US + FNAC resulted in an improved AUC (0.965). Consequently, the addition of a US examination to CT/MRI assisted in the early diagnosis of nodal malignancy in patients with irradiation-treated head and neck cancer.

The combination of CT/MRI with US + FNAC remained the most accurate in terms of the diagnosis of nodal recurrence or persistence. At our institution, FNAC was performed simultaneously when suspicious echogenic findings for malignancy were noted during US studies. These findings included irregular margins, heterogeneous internal echogenicity, the presence of calcification, cystic architecture, absence of echogenic hilum, and a peripheral or mixed vascular pattern [11]. Our results revealed that the false-negative rate was significantly higher in patients who were diagnosed using CT/MRI alone than that of patients diagnosed using CT/MRI combined with US + FNAC (11% [29/269] vs 1% [3/269], *p* < 0.001) during the evaluation of neck LAP. Thus, we suggest not only performing a US examination during follow-up but also obtaining FNAC simultaneously when presented with suspicious ultrasonographic features in the assessment of neck LAP in patients with irradiation-treated head and neck cancer.

**Figure 3:**
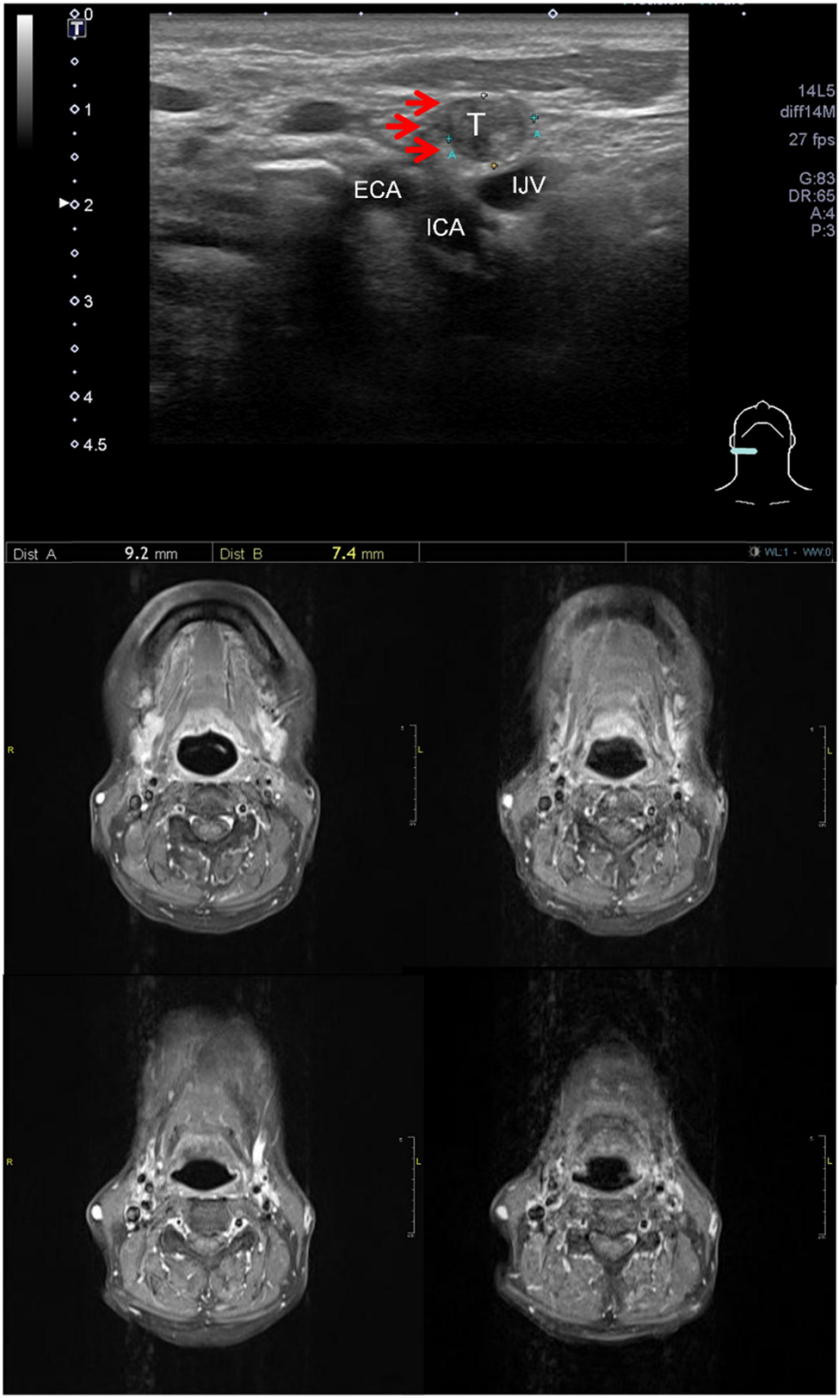
One representative patient. A 59-year-old male was diagnosed with oropharyngeal cancer, stage IVA and received definite CCRT. The follow-up MRI showed no abnormal cervical LN. US showed a round, ill-defined, heterogeneous, and hypoechogenic mass (0.74 cm × 0.92 cm) at right level II (arrow). The cytological and further pathological reports both showed metastatic squamous cell carcinoma. Abbreviations: T, tumor; ECA, external carotid artery; ICA, internal carotid artery; IJV, internal jugular vein.

CT/MRI had a lower NPV than the post-RT US predictive model in our study (78.2% [115 of 147] vs 87.4% [97 of 111]; Table 2). This result may be attributable to post-RT LNs’ tendency to have higher heterogeneity and lower radiodensity in contrast-enhanced CT imaging [15]. Furthermore, our earlier study indicated that the size of recurrent LNs tends to be smaller in patients with a history of RT than that in patients who have never undergone irradiation treatment [16]. Smaller LNs may not be easily detected using a 5 mm cut MRI or 3 mm cut CT. Therefore, a small and less enhanced malignant LN may be classified as benign in a CT/MRI report, generating a false-negative result (Figure 3). The US examination represents a high-resolution continuous imaging study for evaluation of the cervical node [17] and, with the assistance of the predictive model, could produce a higher NPV than CT/MRI.

Although we combined CT/MRI and US + FNAC to increase the diagnostic performance, three false negatives were observed (Table 4). All of these cases involved NPC patients and small LNs (mean [SD] long axis, 0.98 [0.36] cm) of a normal shape (mean [SD] S/L ratio, 0.46 [0.04]). Chan et al. reported a lower NPV (36% vs 74%, *p* = 0.03) and accuracy (54% vs 88%, *p* = 0.05) for FNAC in patients with NPC who had received RT treatment compared with those of patients who were newly diagnosed [18]. A possible reason for this is the histological change of LNs after RT. Cancer cells within LNs might be isolated and unevenly distributed after RT treatment, leading to an increase in false-negative results [8,18]. To address this shortcoming and increase the diagnostic rate for NPC patients, the addition of plasma Epstein–Barr virus (EBV) DNA testing was proposed in one study [18], and the implementation of a combination PET examination during the follow-up period was suggested in another study [8]. Further study may evaluate the diagnostic ability for LAPs when combining the CT/MRI and US + FNAC with plasma EBV DNA or PET.

### 4.1 Limitations

This study has several limitations. First, unnoticed or unavoidable selection bias might have played a role owing to the retrospective study design. Second, this study is based on a convenience sample and we did not calculate the sample size initially. Third, not all our final diagnoses of nodal disease were obtained through pathological diagnosis. Some patients with obvious nodal disease or those that were unsuitable for neck dissection were diagnosed following FNAC and multidisciplinary discussions. Moreover, this study did not identify the most suitable interval at which US imaging studies should be performed. The frequency at which the US study must be conducted and the most cost-effective method requires further evaluation.

## 5 Conclusion

Surveillance of nodal recurrence or persistence is critical during post-RT follow-up in patients with head and neck cancer. Based on this study, CT/MRI combined with either the post-RT US predictive model or US + FNAC had stronger diagnostic performance than CT/MRI alone in assessing nodal malignancy in patients with LAPs treated with irradiation. Postirradiation recurrence or persistent LAPs tend to be more heterogeneous and smaller, which may lead to a lower accuracy rate in evaluations when employing CT/MRI alone. Besides, in this study, the US with CT/MRI was also performed when suspicion of recurrence. Therefore, the results cannot support regular screening over screening on indication. Although this was a retrospective study and the most suitable interval of US examination was not identified, we still recommend performing US studies alongside CT/MRI when suspicion of recurrence increases the early and precise diagnosis of nodal malignancy in patients with irradiation-treated head and neck cancer. Moreover, FNAC can be implemented simultaneously when suspicious ultrasonographic features are detected.

## Supplementary Material

Supplementary material

## References

[1] Burge JS. Histological changes in cervical lymph nodes following clinical irradiation. Proc R Soc Med. 1975;68(2):77–9. 10.1177/003591577506800205 PMC1863641 809772

[2] Rivelli V, Luebbers HT, Weber FE, Cordella C, Gratz KW, Kruse AL. Screening recurrence and lymph node metastases in head and neck cancer: the role of computer tomography in follow-up. Head Neck Oncol. 2011;3:18. 10.1186/1758-3284-3-18 PMC3079696 21439046

[3] Denaro N, Russi EG, Numico G, Pazzaia T, Vitiello R, Merlano MC. The role of neck dissection after radical chemoradiation for locally advanced head and neck cancer: should we move back? Oncology. 2013;84(3):174–85. 10.1159/000346132 23306430

[4] Simo R, Homer J, Clarke P, Mackenzie K, Paleri V, Pracy P, et al. Follow-up after treatment for head and neck cancer: United Kingdom National Multidisciplinary Guidelines. J Laryngol Otol. 2016;130(S2):S208–11. 10.1017/S0022215116000645 PMC4873918 27841136

[5] Zhao X, Rao S. Surveillance imaging following treatment of head and neck cancer. SemOncol. 2017;44(5):323–9. 10.1053/j.seminoncol.2018.01.010 29580434

[6] Jiang H, Tan Q, He F, Yang W, Liu J, Zhou F, et al. Ultrasound in patients with treated head and neck carcinomas: A retrospective analysis for effectiveness of follow-up care. Med (Baltim). 2021;100(16):e25496. 10.1097/MD.0000000000025496 PMC8078385 33879682

[7] Pfister DG, Spencer S, Adelstein D, Adkins D, Anzai Y, Brizel DM, et al. Head and Neck Cancers, Version 2.2020, NCCN Clinical Practice Guidelines in Oncology. J Natl Compr Canc Netw. 2020;18(7):873–98. 10.6004/jnccn.2020.0031 32634781

[8] Toh ST, Yuen HW, Goh YH, Goh CH. Evaluation of recurrent nodal disease after definitive radiation therapy for nasopharyngeal carcinoma: diagnostic value of fine-needle aspiration cytology and CT scan. Head Neck. 2007;29(4):370–7. 10.1002/hed.20526 17123306

[9] Nishimura G, Matsuda H, Taguchi T, Takahashi M, Komatsu M, Sano D, et al. Treatment evaluation of metastatic lymph nodes after concurrent chemoradiotherapy in patients with head and neck squamous cell carcinoma. Anticancer Res. 2012;32(2):595–600. 22287750

[10] Lo WC, Cheng PW, Shueng PW, Hsieh CH, Chang YL, Liao LJ. A real-time prediction model for post-irradiation malignant cervical lymph nodes. Clin Otolaryngol. 2018;43(2):477–82. 10.1111/coa.12998 28981204

[11] Ahuja AT, Ying M, Ho SY, Antonio G, Lee YP, King AD, et al. Ultrasound of malignant cervical lymph nodes. Cancer Imaging. 2008;8:48–56. 10.1102/1470-7330.2008.0006 PMC2324368 18390388

[12] Saito N, Nadgir RN, Nakahira M, Takahashi M, Uchino A, Kimura F, et al. Posttreatment CT and MR imaging in head and neck cancer: what the radiologist needs to know. Radiographics. a review publication of the Radiological Society of North America, Inc. 2012;32(5):1261–82, discussion 1264–82. 10.1148/rg.325115160 22977017

[13] Ritoe SC, de Vegt F, Scheike IM, Krabbe PF, Kaanders JH, van den Hoogen FJ, et al. Effect of routine follow-up after treatment for laryngeal cancer on life expectancy and mortality: Results of a Markov model analysis. Cancer. 2007;109(2):239–47. 10.1002/cncr.22401 17154185

[14] Agrawal A, Hammond TH, Young GS, Avon AL, Ozer E, Schuller DE. Factors affecting long-term survival in patients with recurrent head and neck cancer may help define the role of post-treatment surveillance. Laryngoscope. 2009;119(11):2135–40. 10.1002/lary.20527 19507214

[15] Tang C, Fuller CD, Garden AS, Awan MJ, Colen RR, Morrison WH, et al. Characteristics and kinetics of cervical lymph node regression after radiation therapy for human papillomavirus-associated oropharyngeal carcinoma: Quantitative image analysis of post-radiotherapy response. Oral Oncol. 2015;51(2):195–201. 10.1016/j.oraloncology.2014.11.001 PMC4502963 25444304

[16] Lo WC, Cheng PW, Wang CT, Shueng PW, Hsieh CH, Chang YL, et al. The effect of radiotherapy on ultrasound-guided fine needle aspiration biopsy and the ultrasound characteristics of neck lymph nodes in oral cancer patients after primary treatment. PLoS One. 2016;11(3):e0149346. 10.1371/journal.pone.0149346 PMC4783113 26954569

[17] Na DK, Choi YJ, Choi SH, Kook SH, Park HJ. Evaluation of cervical lymph node metastasis in thyroid cancer patients using real-time CT-navigated ultrasonography: preliminary study. Ultrasonography. 2015;34(1):39–44. 10.14366/usg.14030 PMC4282224 25327528

[18] Chan JY, Chan RC, Chow VL, To VS, Wei WI. Efficacy of fine-needle aspiration in diagnosing cervical nodal metastasis from nasopharyngeal carcinoma after radiotherapy. Laryngoscope. 2013;123(1):134–9. 10.1002/lary.23373 22907783

